# Convergence in floodplain pond communities indicates different pathways to community assembly

**DOI:** 10.1007/s00027-023-00957-9

**Published:** 2023-03-31

**Authors:** P. C. M. Chanut, F. J. Burdon, T. Datry, C. T. Robinson

**Affiliations:** 1 Department of Aquatic Ecology, Eawag, 8600 Duebendorf, Switzerland; 2 Institute of Integrative Biology, ETH-Zurich, 8092 Zurich, Switzerland; 3 Te Aka Mātuatua - School of Science, University of Waikato, Hamilton, New Zealand; 4 INRAE, UR RiverLy, Centre de Lyon-Villeurbanne, Villeurbanne, France

**Keywords:** Floodplain, Metacommunity, Macroinvertebrates, Succession, Disturbance

## Abstract

Disturbance can strongly influence ecosystems, yet much remains unknown about the relative importance of key processes (selection, drift, and dispersal) in the recovery of ecological communities following disturbance. We combined field surveys with a field experiment to elucidate mechanisms governing the recovery of aquatic macroinvertebrates in habitats of an alluvial floodplain following flood disturbance. We monitored macroinvertebrates in 24 natural parafluvial habitats over 60 days after a major flood, as well as the colonization of 24 newly-built ponds by macroinvertebrates over 45 days in the same floodplain. We examined the sources of environmental variation and their relative effects on aquatic assemblages using a combination of null models and Mantel tests. We also used a joint species distribution model to investigate the importance of primary metacommunity structuring processes during recovery: selection, dispersal, and drift. Contrary to expectations, we found that beta diversity actually decreased among natural habitats over time after the flood or the creation of the ponds, instead of increasing. This result was despite environmental predictors showing contrasting patterns for explaining community variation over time in the natural habitats compared with the experimental ponds. Flood heterogeneity across the floodplain and spatial scale differences between the experimental ponds and the natural habitats seemingly constrained the balance between deterministic and stochastic processes driving the ecological convergence of assemblages over time. While environmental selection was the dominant structuring process in both groups, biotic interactions also had a prominent influence on community assembly. These findings have profound implications towards understanding metacommunity structuring in riverscapes that includes common linkages between disturbance heterogeneity, spatial scale properties, and community composition.

**Supplementary Information:**

The online version contains supplementary material available at 10.1007/s00027-023-00957-9.

## Introduction

Determining the processes that structure communities is a central goal in ecology (Logue et al. 2011). Two alternative models have traditionally been used to examine community structuring. The niche-based model states that abiotic conditions and biotic interactions ‘filter’ species, thus determining community composition (Chase and Leibold 2004). In contrast, the neutral model considers all species equivalent and states that the inherent stochasticity in the realization of vital rates (birth, death and extinction) or dispersal determine community composition (Bell 2001; Hubbell 2001). These contrasting views have been reconciled where deterministic and stochastic forces act simultaneously, with differing relative importance depending on the context (Gravel et al. 2006; Thompson et al. 2020).

The traditional metacommunity concept provides a useful framework to study how the interplay between stochasticity and determinism shapes communities (Leibold et al. 2004). Vellend (2010, 2016) proposed four major processes in metacommunity structuring: selection, drift, dispersal and diversification. In community ecology, selection describes the deterministic filtering of species according to their respective fitness within different abiotic and biotic environments. A distinction is made between constant selection that describes selection of species based on fixed fitness differences within a given environment, and density-dependent selection where the fitness of a species also varies with its own density and possibly with that of other species. When the fitness of a species varies with its density, the chronological order of colonization can have far reaching effects on community composition, giving rise to priority effects, where early colonists can rapidly occupy or modify the niches of other species (Almany 2003; Fukami 2015; Vellend 2016). Drift represents the stochastic component of community trajectory, resulting from inherent stochasticity in the realization of vital rates within populations. Importantly, the outcomes of selection and drift may vary with dispersal (Chase 2003; Vellend 2010, 2016). For instance, high dispersal rates can give rise to mass effects, thereby overriding species–environment relationships (Leibold et al. 2004; Holyoak et al. 2005) and dispersal limitation may limit the ability of species to track environmental variation, thus favouring drift (Chase 2003; Fernandes et al. 2009; Dias et al. 2016). Lastly, diversification refers to the generation of new species and often is omitted from metacommunity studies, given the long temporal scale typically associated with this process.

The relative importance of these processes is strongly linked to environmental context, and characterizing these linkages is crucial to the understanding of metacommunity structuring. For instance, stochastic processes, such as drift, are more likely to be important in productive habitats and in low-disturbance settings (Chase 2003, 2007). The importance of drift also tends to increase for smaller and more isolated communities (Chase 2003; Lande et al. 2010; De Meester et al. 2016). Similarly, younger, less mature communities are more likely to be dominated by stochastic processes, such as dispersal than mature communities (Jenkins 2006; Larsen and Ormerod 2014). Finally, the role of dispersal on community structure depends on the degree of landscape connectivity (or spatial scale) with respect to the dispersal abilities of the taxa found in the regional species pool (Thompson and Townsend 2006a; Cañedo-Argüelles et al. 2015; Heino et al. 2015; Datry et al. 2016).

Riverine floodplains are highly dynamic and heterogeneous ecosystems, making them ideal systems to study metacommunity structuring (Tockner et al. 2010). Floodplains are commonly described as shifting habitat mosaics, because frequent flooding ensures high spatio-temporal turnover in floodplain habitats and connectivity patterns (Stanford et al. 2005). In addition, communities undergo frequent disturbance–recovery cycles during and after floods, which gives particular importance to recovery and succession processes in maintaining biodiversity patterns. Environmental variation among aquatic habitats in floodplains mostly results from their location within the geomorphological setting, which determines the origin of waters (e.g. hillslope runoff, shallow or deep groundwater, tributaries) and their degree of hydrological connectivity (HC). Lateral HC through surface water connection with the main stem, vertical and longitudinal HC through connection to groundwater, and hyporheic flow paths are primary drivers of environmental and biotic diversity (Amoros and Bornette 2002; Opperman et al. 2010; Capderrey et al. 2013). For instance, highly connected aquatic habitats exhibit physico-chemical conditions resembling the river channel and favour rheophilic taxa (Amoros and Bornette 2002; Brunke et al. 2003; Larned and Datry 2013).

Floods usually have a homogenizing effect within riverscapes, and many floodplain studies observed that environmental and biotic heterogeneity decreased during or immediately after floods and increased during periods of hydrological isolation, leading to convergence–divergence patterns in beta diversity (Thomaz et al. 2007; Sarremejane et al. 2017; Van Looy et al. 2019). This homogenization has been associated with a decrease in heterogeneous deterministic selection (Larsen et al. 2019), and also can result from increased dispersal during floods (Frisch and Threlkeld 2005; Petsch et al. 2017; Nicacio and Juen 2018; Chen et al. 2022). However, the majority of floodplain studies (but see Larsen et al. 2019) were conducted on large river floodplains where floods resemble more slowly advancing inundation fronts than the hydrodynamic disturbances that affect mid-sections of most European rivers (Tockner et al. 2000; Larsen et al. 2019). Further, most studies examined community structuring processes and infer their dependency to the environmental context (disturbance attributes, spatial scale) of a given system. Here, we argue that by allowing the spatial scale, degree of ecological connectivity and environmental heterogeneity to vary within the same floodplain, and compare findings among aquatic habitats, novel insights can be gained on the linkages between environmental context and community structuring.

In this study, we combined repeated field surveys and a field experiment to investigate how the environmental setting (spatial scale, disturbance attributes, environmental heterogeneity) influences metacommunity processes. Specifically, we monitored macroinvertebrate assemblages in 24 floodplain aquatic habitats over 60 days after a major flood in summer 2016 and, in parallel, we created 24 experimental ponds in the floodplain and examined assemblages over 45 days after construction. We expected that the creation of novel ponds would be analogous to a homogeneous flood disturbance, resetting pond assemblages to a similar initial successional stage. This expectation was because all experimental ponds were constructed on the same day. Although initial physico-chemical conditions might differ, we predicted that all ponds would start off with very few individuals, making dispersal from other sources essential for colonization and subsequent community assembly. To investigate these predictions, we first characterized the environmental differences among floodplain aquatic habitats, their causes, and their influence on assemblages. Second, we examined how temporal shifts in the balance between deterministic selection and stochasticity explained temporal patterns in beta-diversity. Finally, we investigated the relative importance of metacommunity processes and their likely linkages to spatial scale, disturbance and environmental context. We hypothesized that environmental variation among habitats would primarily result from differences in HC, and this variation would initially be low, reflecting the homogenizing effect of the flood (or habitat creation) (H1). We expected that beta diversity would be low initially and increase overtime (H2). This prediction would reflect the fact that experimental ponds would start from a homogeneous ‘baseline’ successional stage with a few individuals, while the flood homogenized natural communities by allowing dispersal of individuals (via lateral HC). Over time, deterministic selection would increase with diverging environmental conditions acting as a filter on communities in both systems (experimental and natural ponds). On average, over the whole study period, we expected that selection would be stronger in the natural habitats because of greater environmental heterogeneity (where habitats with high HC would favour rheophilic taxa) and spatial scales influencing aerial dispersal, whereas more stochastic processes such as drift, mass and priority effects would be relatively more important in the experimental ponds (H3).

## Methods

### Study area

**Fig. 1:**
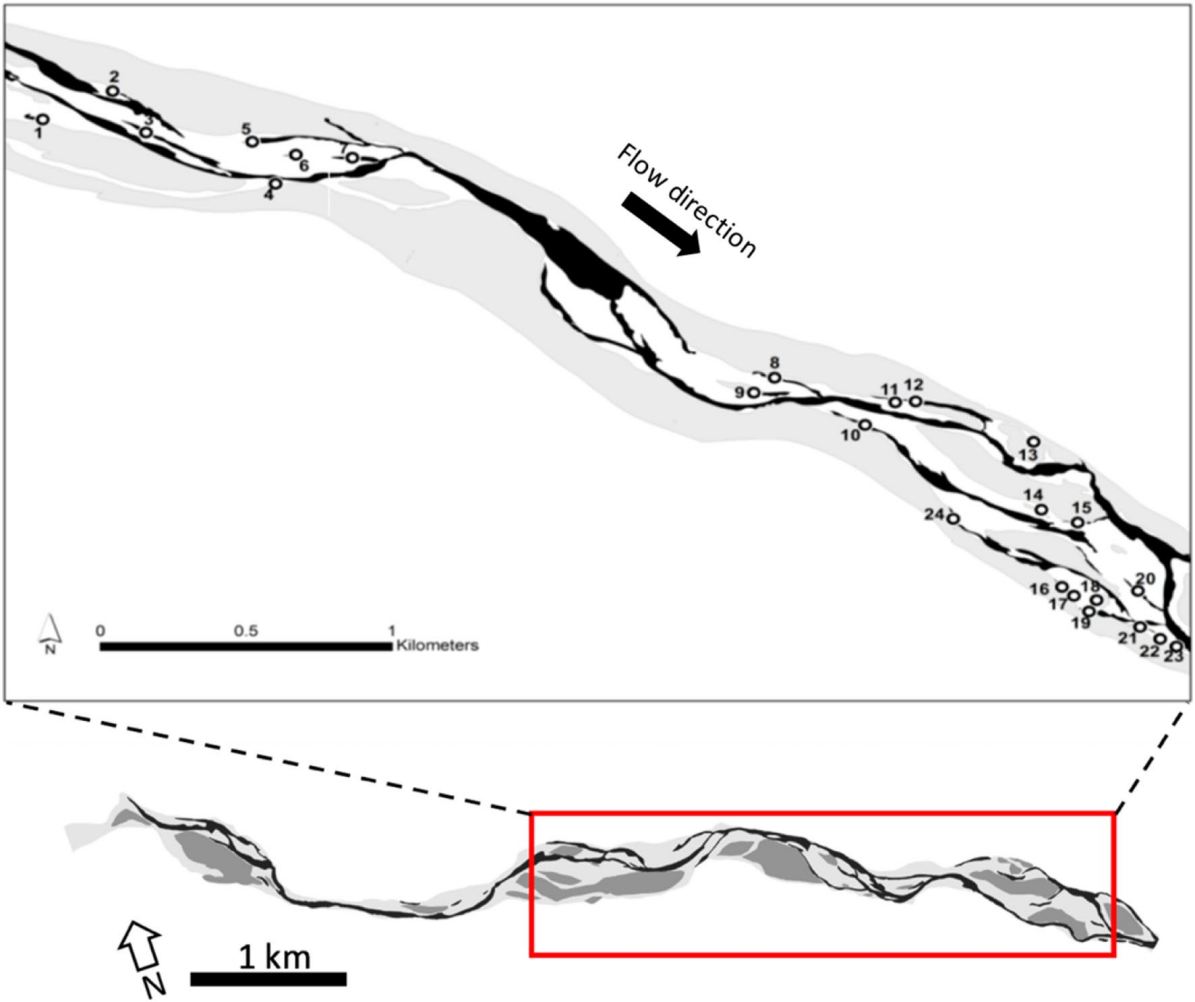
Map showing the Maggia River floodplain in the Swiss canton of Ticino. The inset indicates the location of ‘natural’ sites surveyed in the floodplain

The Maggia River is located in the southern part of the Swiss Alps and possesses one of the last remaining natural floodplains in Switzerland. The Maggia River catchment covers an area of 930 km^2^ with elevations ranging from 200 to 3300 m a.s.l. Originally, the Maggia was characterized by a glacio-nival hydrological regime. But since 1953 and the construction of a hydropower scheme in the headwaters, discharge is mostly constant (~ 1.5 m^3^/s at the upstream end of the floodplain), except for occasional flood peaks occurring with uncorrelated magnitude and return periods (Perona et al. 2009). The alluvial floodplain used in this study has an elevation of about 350 m a.s.l., is about 7 km long and nearly 2 km wide in some sections. The combination of high flows and abundance of bedload sediment maintains a diverse floodplain mosaic, where various types of parafluvial habitats occur within braided sections. For this study, we selected 24 permanently-wetted parafluvial habitats encompassing permanent ponds, disconnected side arms, and side arms connected at their downstream end with a shallow riffle (Fig. 1). Importantly, a relatively large flood occurred on 16 June 2016 (discharge = 478 m^3^/s, return period = 0.79) that affected all natural parafluvial habitats in the study area.

### Experimental setup

**Fig. 2:**
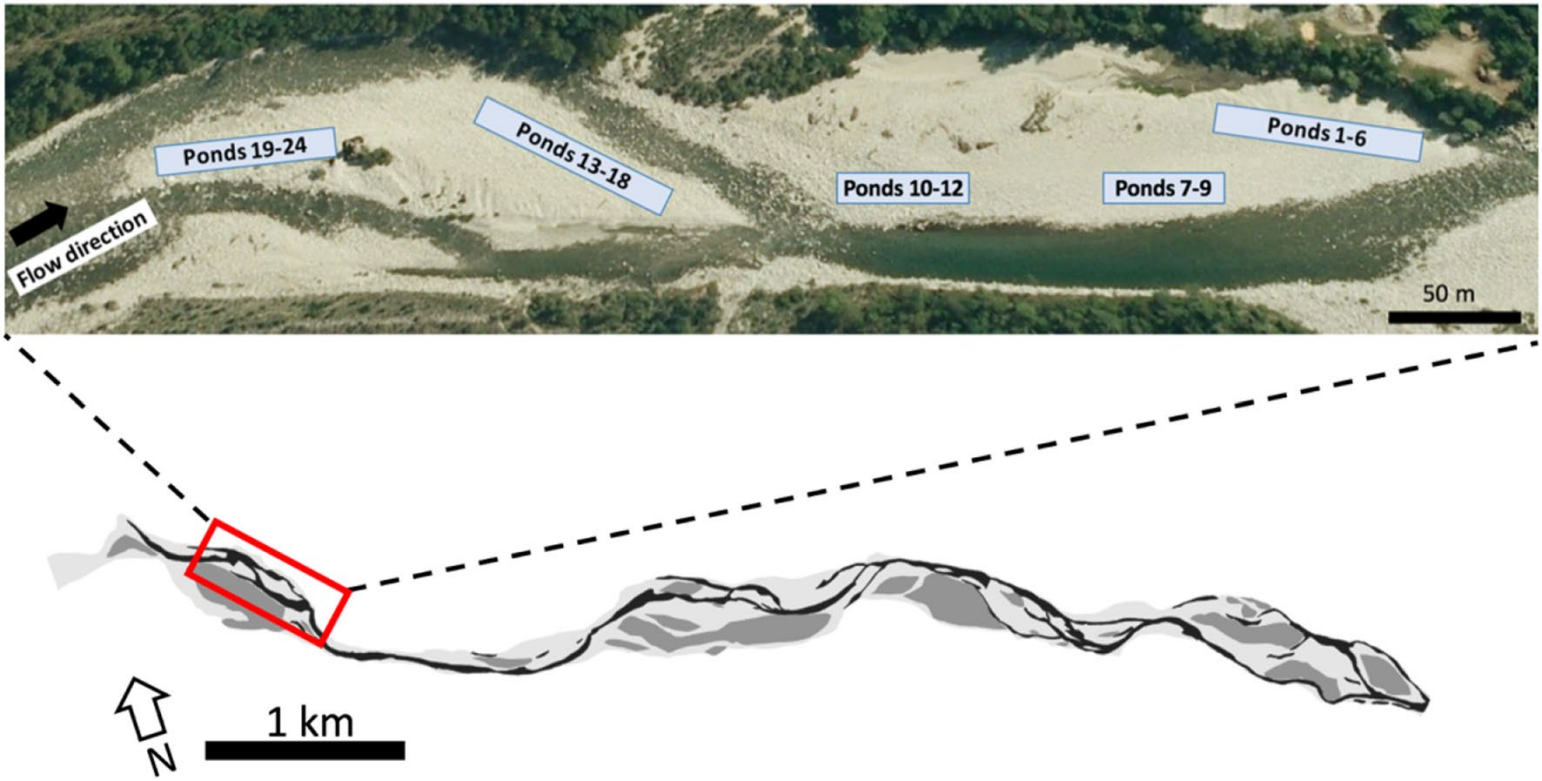
Map showing the location of the experimental ponds in the Maggia River floodplain

Approximately 2 km upstream from the last sampled natural habitat, on July 14, we excavated 24 ponds (of approximate 2 m × 2 m dimensions) in two gravel bars of the floodplain using a mechanical digger (Figs. 2, and S1.1). The constructed ponds were distributed within the active floodplain (i.e. the area around a river regularly flooded on a periodic basis), with 12 ponds located on one gravel bar, and 12 ponds on another bar about 200 m upstream on the opposite bank. Because the ponds were filled by exfiltration of hyporheic (i.e. shallow subterranean) water from the river, we anticipated that pond location along the upstream–downstream direction on each gravel bar would influence the length of hydrological flow paths, and thus their degree of HC to the main river channel, thereby affecting their physico-chemical properties (Lowell et al. 2009; Boulton et al. 2010; Larned and Datry 2013). In this context, six ponds were clustered at the downstream end and six at the upstream end of each gravel bar. In each of these clusters, ponds were also distributed along an upstream–downstream axis. Ponds were distributed in clusters where average, minimum and maximum distances between ponds were 153 m, 3 m and 322 m, respectively.

The aim of the experiment was to recreate a system of ponds that would be directly comparable to the natural parafluvial habitats, but differ from it in terms of spatial scale and disturbance history. Comparing the two systems would therefore allow use to directly assess the effects of these two variables on the structuring of metacommunities. Here, there were several factors that made the natural habitats and the experimental ponds comparable. First, the experiment was spatially embedded in the natural floodplain, which meant that the taxa pool for potential colonization was likely common to both systems. Second, sampling in the two systems was largely overlapping in time, implying that the different taxa were sampled at similar life stages in the two systems, which is critical when dispersal primarily occurs with aerial adult stages. On the other hand, the distances between the experimental ponds were much smaller than among floodplain habitats, which enabled us to assess the effects of spatial scale on metacommunity structuring. In addition, ponds were constructed on the same day and were very similar in their habitat conditions and communities at the start of the study, whereas natural parafluvial habitats were recently affected by a natural flood. We could therefore compare the structuring of metacommunities between two disturbance types, with the experimental ponds mimicking the homogenizing effect of a prolonged inundation and the natural system of a more pulsed and spatially-heterogeneous flood.

### Sampling and laboratory analysis

Sampling sites in the floodplain were monitored on four dates after the flood: June 27, July 11, July 27 and August 15. In subsequent analyses, these dates are labelled as Day 11, 25, 41 and 60, respectively, reflecting the time elapsed since the flood. On each date, a water sample (0.5 L) was collected from each habitat and analysed for nitrate (NO_3_, mg/L), silicate (mg/L), and pH using standard methods following Tockner et al. (1997). Spot measurements of dissolved oxygen (mg/L, Hach HQ40d connected to a LD10101 oxygen probe), water temperature (°C) and electrical conductivity (µS/cm, WTW meter, Germany) were taken on each visit. Electrical conductivity, silicate and nitrate concentrations are useful indicators for hydrological connectivity because these may be more elevated in groundwaters.

Biofilms were measured by randomly selecting five stones (cobble-size). Biofilm was removed from each rock by scrubbing with a wire brush into a plastic container with 100 mL distilled water, and the scrubbed area measured (Uehlinger 1991). The biofilm suspension was subsequently filtered through a glass fibre filter (0.45 μm, Whatman GFF) and stored on ice in the dark. We split the filters in half and used each half to quantify ash-free dry mass (AFDM) and chlorophyll-a concentration, respectively, as proxies for biofilm standing crop. For estimates of biomass as AFDM, half filters were dried at 60 °C for 24 h, weighed, then combusted at 450 °C for 6 h and reweighed. For chlorophyll-a extraction, the other half filter was incubated in 6 mL 90% ethanol at 70 °C for 10 min. Chlorophyll-a (µg /L) was then determined using spectrophotometry (Hitachi 2000) following methods in Meyns, Illi and Ribi (1994).

Benthic particulate organic matter (POM) was collected at three locations within each habitat using a Hess sampler (250 µm mesh, 0.04 m^2^ area) by disturbing the substrate at a depth of ~ 10 cm. The collected POM was frozen (−20 °C) until analysis. Once in the laboratory, POM was quantified as AFDM, as done for biofilm. In each habitat, substrate size composition was measured using a zig-zag pebble count method (100 stones were measured). Benthic macroinvertebrates were randomly collected (*n* = 3 at each site and date) using a Hess sampler (250 µm mesh, 0.04 m^2^ area) and preserved in 70% ethanol.

In the experimental pond system, sampling occurred on July 29, July 20, August 13 and 28 August 2016, or day 15, 20, 30 and 45 after building the ponds, respectively. This period of 45 days is longer than the average return period of 33 days for flows above 20 m^3^/s (between 2006 and 2016), which have been observed to inundate parafluvial habitats in the Maggia floodplain (data from the Swiss Federal Office for the Environment). The order in which sampling sites were monitored in the experimental and natural systems was randomized at each sampling event to reduce bias of collection time. On each visit, a water sample (0.5 L) was collected from the centre of each experimental pond and analysed for the same elements as in the natural system. Spot measurements of dissolved oxygen water temperature (°C) and electrical conductivity were also taken on each visit. Biofilm collection and quantification was completed with the same method as in the natural system, but here chlorophyll-a concentration was used as a proxy for biofilm standing crop.

We used a kick-net (250 μm mesh) to sample macroinvertebrates and benthic POM in the experimental ponds, where a standard surface area (~ 90 cm^2^) was disturbed for 30 s and the net pulled 10 times through the suspended material. This method is not as strictly quantitative as the Hess sampler, but is more suitable to the sampling of standing waters (Oertli et al. 2005). A different section of each pond was sampled on each sampling event to minimize disturbance effects. This method was suitable for collecting macroinvertebrates as substrate size and benthic primary production in each pond appeared relatively homogeneous. Collected POM was analysed in the lab using the same method as for natural habitats. Substrate size composition was not assessed in the experimental ponds because this involved walking in an extensive portion of the ponds, which would have been too destructive considering the small size of these ponds in comparison with the natural habitats.

In the laboratory, macroinvertebrate samples from both systems were processed using the same method. Collected individuals were hand-picked, counted and identified using Tachet et al. (2010). In general, 65% of the taxa were identified at the family level and 45% at the genus level. Recent research showed that higher taxa (genera and families) performed well for studying community composition when niche conservatism is high within higher taxa (Rosser 2017), and at small spatial scales where the ratio of species to higher taxa is low (SHR < 2–3; Heino and Soininen 2007; Carneiro Bini and Rodrigues 2010; Heino 2011; Timms et al. 2013). Here, the spatial scale of our study is very small compared with the distribution range of our taxa, the SHR is thus likely to be low, as also found in studies of other rivers in the region (Brunke et al. 2003; Chanut, unpublished data). In addition, many studies have shown that linkages between environment and community composition in stream macroinvertebrates were well preserved across species, genera and families, reflecting the high niche conservatism within these higher taxa (Furse et al. 1984; Arscott et al. 2006; Beketov et al. 2009; Datry et al. 2014). We therefore concluded that using families and genera to describe community composition was adequate in this study.

### Data analysis

In both systems, we used proxies to quantify vertical HC. Specifically, we assumed that hyporheic flow paths were parallel to the main flow direction, and that infiltration zones would be located primarily at the upstream tip of the gravel bars on which natural and experimental habitats were located. We therefore used the distance from a given habitat to the upstream tip of the gravel bar as a proxy for vertical HC, and assumed that HC would decrease in an upstream–downstream direction within each gravel bar. It must be noted that this method is only an approximation. Hyporheic flow paths are more complex than upstream–downstream surface flows, because belowground flows may preferentially follow former channels buried in the riverbed. The heterogeneity of flow paths means gravel bars may receive water from different origins and thus vary in water compositions. We also used the longitudinal coordinate (*Y*) to describe the location of aquatic habitats along the floodplain, and thus account for different origins of water and geomorphological differences among gravel bars that may affect the physico-chemical environment of aquatic habitats. This was particularly relevant because downstream reaches of the Maggia floodplain are typically gaining reaches, and could therefore differ from upstream losing reaches in terms of hydrological processes and water composition.

To evaluate our predictions, we performed a principal component analysis (PCA) on all measured environmental variables (after removing highly correlated variables, see Table S1.1) to decompose information into major environmental gradients. We then used generalized additive mixed models (GAMMs) to assess how the first PCA axes varied with the distance from the upstream tip of the gravel bars, *Y* coordinate and temporal attributes. For temporal attributes, we used the time since last flood in the natural system and the time since the construction of the ponds in the experiment. Following Siebers et al. (2019), the number of knots in all GAMMs was limited to 4, to avoid over-interpolation. Site identification was also added as a random effect in all models to account for temporal replication.

In both systems, spatial taxonomic beta diversity was calculated at each date using the Bray–Curtis index on the basis of log-transformed abundance data. In all diversity analyses, we chose to use abundances over occurrences, because abundance differences are likely to yield insightful results and allow a clearer discrimination between metacommunity processes (Anderson et al. 2011; Ovaskainen et al. 2017). To estimate the relative importance of deterministic and stochastic structuring forces on the communities, we used a combination of two approaches. First, we compared the temporal variations in environmental heterogeneity (between habitats), beta diversity and beta deviations (or the difference between observed and randomly expected beta diversity). Environmental heterogeneity was measured as the average of all pairwise Euclidean distances on the basis of the environmental variables used in the PCAs (Table S1.3). Beta diversity was measured as the mean pairwise Bray–Curtis distances among communities. Finally, following Chase and Myers (2011) and Kraft et al. (2011), we used a null model approach (Tucker et al. 2016) to compare observed beta diversity with the null expectation. Beta deviations are closer to 0 when stochastic processes dominate, whereas shared environmental filtering across communities should make beta deviations more negative, and strong biotic interactions or selection within a heterogeneous environment should drive positive beta deviations. In addition, priority effects resulting from stochastic colonization, followed by deterministic biotic interactions, can lead to alternative community compositions and therefore high beta deviations, in otherwise similar environmental conditions (Chase 2003; Chase and Myers 2011).

In addition, following Cañedo-Argüelles et al. (2020), we used Mantel tests to estimate the importance of selection and dispersal on community composition. Importantly, this analysis did not attempt to partition the variation in community dissimilarity between specific processes [using the Hierarchical Modelling of Species Communities (HMSC) framework, see below], but rather to broadly assess the temporal changes in the amount of explained variation, thus the putative strength of the deterministic component. Community dissimilarities were calculated with the Bray–Curtis index. Following Cañedo-Argüelles et al. (2020), we used classical Mantel tests to test the effects of Euclidean spatial distances, and Mantel tests corrected for spatial autocorrelation (MSR) to test the effect of Euclidean environmental distances, on community dissimilarity (Crabot et al. 2019).

Finally, we used the Hierarchical Modelling of Species Communities (HMSC) framework (Ovaskainen et al. 2017) as a variation partitioning method to estimate the relative influence of key metacommunity processes on community structure. This framework has been successfully used to disentangle metacommunity processes in observational studies (Stark et al. 2018a, b; Chiu et al. 2020) and provides a robust alternative to the more classical partial redundancy analysis prone to producing inflated R^2^ and overestimating or underestimating environmental and spatial components (Gilbert and Bennett 2010; Tuomisto et al. 2012). HMSC is a joint species distribution model, meaning that it models the effects of a set of explanatory variables on all populations of the community simultaneously; these are then aggregated to estimate community-wide responses (Tikhonov et al. 2017). Because our main question related to the overall relative importance of metacommunity processes in each system, we conducted the analysis on all dates together. This method also increased the power of the models owing to the greater sample size, and allowed us to explicitly model the effect of time on communities. The environmental variables used in the PCAs were included as fixed effects in the models and following Ovaskainen et al. (2017), spatial structure was described as random effects. To reduce multicollinearity, we calculated the variance inflation factor (VIF) for the model and removed all variables with VIF > 3. We included sampling sites as a random effect in both systems. The variables ‘time since flood’ or ‘time since creation of the ponds’ were added as fixed effects to account for temporal variation in species abundances independent of other covariates, which could reveal drift or succession patterns.

To test for the prevalence of priority effects or strong biotic interactions, we used log-transformed abundances of the most abundant taxa at the first sampling date as fixed effects (Ovaskainen et al. 2017; Little and Altermatt 2018). We used default priors and Poisson likelihood distributions for abundance data, and ran 100,000 iterations of the MCMC chains, a burn-in period of 1000 iterations and thinned to every 100th sample of the posterior distributions. The full model (SPE) included environmental (E) fixed effects as well as spatial (S) random effects and the abundances of the most abundant taxa at previous dates (P). Following Little and Altermatt (2018), we compared the R^2^ of several models to select the best fitting one, including the SPE model and different combinations of environmental, spatial and biotic variables (SE, SP, S, see Table S1.4). Because the first date was removed from the analysis to include the effects of previous abundances, a model including the effects of spatial and environmental variables on all dates (SE_full_) was also examined (Little and Altermatt 2018). All analyses were completed with R4.0.3 (R core team 2020). We used the *mgcv* package for GAMMs, *vegan* for betadiversity calculations, *ade4* for Mantel tests, *adespatial* for MSR and *Hmsc* for the HMSC models.

## Results

### Location and disturbance determine environmental variation among habitats

The first PCA axis (PC1) explained 34% of the environmental variation in the experiment and 36% in the natural system. In both systems, PC1 appeared to represent differences in hydrological connectivity (HC), where poorly connected habitats with high electrical conductivity and high chlorophyll-a concentrations were described with negative axis scores, whereas strongly connected habitats showed high dissolved oxygen (DO) levels and were described with positive axis scores (Table S1.1, Figs. S1.2 and S1.3).

In the experiment, PC1 decreased along the distance from the upstream tip of the gravel bar and over time, and had a positive parabolic relationship with the *Y* coordinate (Figs. S1.4, S1.5, Table S1.2). In the natural system, PC1 decreased with increasing distance from the upstream tip of the gravel bar and along the *Y* coordinate (increasing in a downstream direction) (Fig. S1.6). Although the second PCA axes explained around 20% of the variation in both systems, their interpretation was less clear; thus, these axes were not used in the following analyses.

### Temporal changes in beta diversity, beta deviations and environmental heterogeneity

In both systems, taxonomic beta diversity (TBD) appeared to slightly decrease over time, suggesting a moderate structural convergence among communities (Table 1). Beta deviations were positive and also seemed to decrease slightly over time, suggesting that communities were overall more dissimilar than expected by chance, but became slightly more stochastic over time. The degree of environmental heterogeneity between habitats increased over time in the experiment, whereas it decreased in the natural system (Table 1).

**Table 1:** Table presenting the mean values (with standard deviations) for environmental heterogeneity (pairwise Euclidean distances calculated on habitat conditions), beta diversity (pairwise Bray–Curtis dissimilarities) and beta deviations at each sampling date for the experiment and natural system

	Days	Environmental heterogeneity	Beta diversity	Beta deviation
Experiment	15	3.53 (1.22)	0.4 (0.13)	0.32
	20	3.49 (1.36)	0.39 (0.11)	0.31
	30	3.52 (1.27)	0.36 (0.12)	0.29
	45	3.53 (1.25)	0.34 (0.12)	0.28
Natural	11	4.83 (1.41)	0.45 (0.13)	0.33
	25	3.93 (1.87)	0.42 (0.12)	0.30
	41	3.73 (1.57)	0.40 (0.13)	0.29
	60	3.53 (1.35)	0.35 (0.12)	0.25

### Temporal changes in environment and spatial influences on community structure

**Fig. 3:**
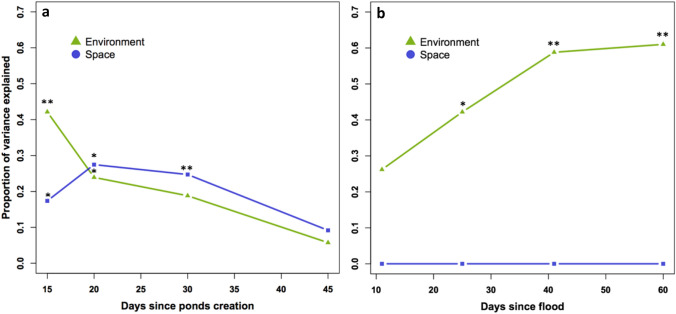
Mantel tests showing the effect of environmental and spatial distances on community dissimilarity in the experiment (a) and in the natural system (b). Stars represent the significance level of the tests (*p < 0.05, **p < 0.01, ***p < 0.001)

Mantel tests showed that, for the experiment, the effect of environmental distance on community dissimilarities was highest at day 15 and decreased over time until day 30 when its effect was no longer significant. In contrast, the effect of spatial distances in the experiment increased between day 15 and day 20, then decreased slightly at day 30, and was no longer significant at day 45 (Fig. 3a). In the natural system, the effect of environmental distance was non-significant at day 11, then a significant effect was high and increased between day 25 and day 60. In contrast, the effect of spatial distance was non-significant at all dates (Fig. 3b).

### The relative importance of metacommunity processes

**Fig. 4:**
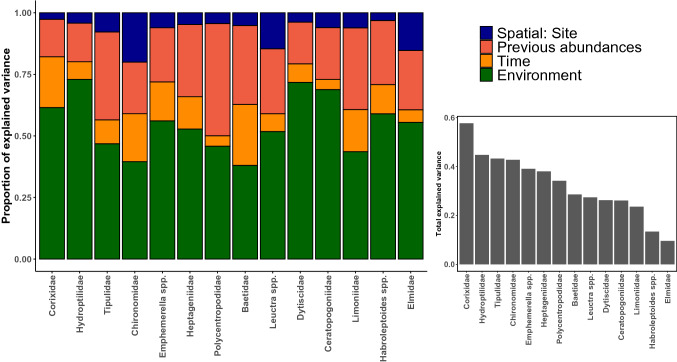
Results of the HMSC model for the natural system, showing the total amount of variation explained by the model for each taxon, as well as the proportion of variation explained by each predictor category

In both systems, the model comparison showed that the full SPE model explained the most variation in species abundances with R^2^ of 0.33 and 0.39 for the natural system and the experiment, respectively (Table S1.4). In the natural system, the environment contributed the most to the explained variation in taxa abundances (55% on average, Fig. 4). In particular, the distance to the upstream tip of the gravel bar had negative associations with 9 out of 14 taxa (Table S1. 6). DO concentrations had a positive association with the abundances of Hydroptilidae and negative associations with Ceratopogoniidae, Corixidae and *Ephemerella* spp. Finally, silicate concentrations had positive associations with Corixidae and Dytiscidae, and a negative association with Hydroptilidae. Previous abundances of dominant taxa were the second strongest predictor, accounting for on average 25% of explained variation, but no interaction was particularly strong here except the positive association between abundances of Tipulidae and their previous abundances (Fig. 4, Table S1.5). The time elapsed since the flood accounted for on average 12% of the explained variation and was positively associated with Baetidae, Corixidae and Dytiscidae. Finally, the random effect describing the spatial structure among sites only accounted for, on average, 7.4% of the explained variation.

**Fig. 5:**
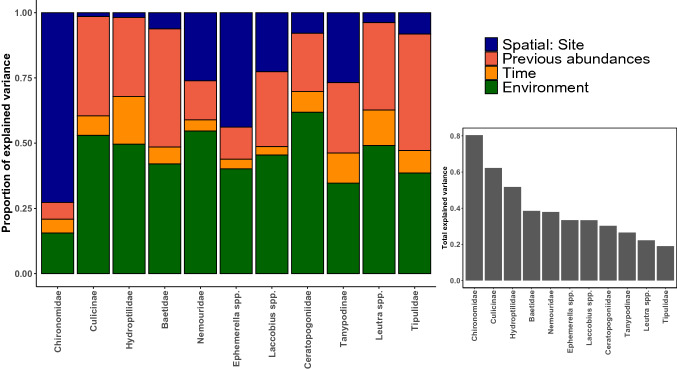
Results of the HMSC model for the experiment, showing the total amount of variation explained by the model for each taxon, as well as the proportion of variation explained by each predictor category

In the experiment, environmental variables explained on average 44% of the variation, with DO and chlorophyll-a affecting the most taxa (Fig. 5). In particular, ponds with low-HC conditions characterized by high chlorophyll-a and low DO had higher abundances of Chironomidae, Culicinae, Hydroptilidae and *Laccobius* spp., while high-HC conditions favoured Baetidae, Ceratopogoniidae, *Ephemerella* spp. and Nemouridae (Table S1.6). Here, previous abundances of dominant taxa accounted for 27.6% of the total variance explained. We observed that abundances of Culicinae, Baetidae and Tipulidae were positively associated with their previous abundances. The spatial location of ponds had a moderate effect overall with an average of 20.1%, but this effect was strongest for Chironomidae, *Ephemerella* spp. and Tanypodinae (75.7%, 44.5% and 23.1%, respectively). Finally, the time since pond creation only accounted for 6.2% of the explained variation and was negatively associated to the abundances of Hydroptilidae.

## Discussion

Our research addressed knowledge gaps regarding the relative importance of different structuring processes in metacommunities of floodplains. These gaps in our understanding include the temporal dynamics of the metacommunity processes following disturbance and habitat creation. We compared metacommunities between natural floodplain parafluvial habitats and experimental ponds to study how the environmental, spatial and disturbance contexts affected the relative importance of structuring processes. We found that environmental variation among floodplain parafluvial habitats depended largely on their degree of vertical hydrological connectivity (HC) and origins of water linked to their positioning within the floodplain. Contrary to expectations, we found that the flood increased heterogeneity among habitats and communities. This contradiction was likely because the flood acted more as a spatially-heterogeneous disturbance event than as a homogenizing pulse of lateral connectivity. Importantly, we showed that although there was a moderate convergence among communities in both systems, this reduction in beta-diversity apparently resulted from different processes. Specifically, selection along re-forming environmental gradients caused the convergence in the natural system, while mass effects occurred in the experiment because of the small spatial scale, thus homogenizing communities.

### Hydrological connectivity determines environmental variation among floodplain habitats

In the two systems, differences in vertical HC and the origins of water were the main sources of environmental variation among habitats. Habitats with high vertical HC were located closer to the upstream tip of gravel bars. Environmental conditions in these high-HC habitats are relatively similar to the main channel because of the high inflow of recently infiltrated surface water. In contrast, low-HC habitats hold proportionally more older groundwater, characterized by high electrical conductivity and low oxygen, as well as high nutrient concentrations that boost primary productivity (Brunke and Gonser 1997; Brunke et al. 2003; Lowell et al. 2009; Larned and Datry 2013; Boulton et al. 2017).

The differences in water origin were more apparent when examining environmental conditions along the longitudinal dimension of the floodplain (*Y* coordinate). The parabolic response of PC1 along the *Y* coordinate in the experiment highlighted the complexity in hydrology, showing that environmental heterogeneity among ponds resulted from a combination of differences in HC and water origin (Brunke and Gonser 1997; Amoros and Bornette 2002; Brunke et al. 2003). Similarly, there were large scale variations in water origin within the floodplain, where elevated silicate concentrations in downstream habitats indicated upwelling of older groundwater (Ruf et al. 2008). In the natural system, the temporal trends of decreasing DO, and increasing silicate concentrations and primary production reflect the receding effect of the flood. Floods rejuvenated environmental conditions across the floodplain, and parafluvial habitats then evolved towards low-HC conditions (with decreasing DO, increasing productivity and electrical conductivity) during the ensuing phase of hydrological isolation (Amoros and Bornette 2002; Thomaz et al. 2007).

We observed temporal changes in environmental heterogeneity within both systems. Specifically, environmental conditions were initially homogeneous and gradually diverged among ponds in the experiment, while doing the opposite in the natural system. This suggests that the flood created environmental heterogeneity among floodplain habitats, likely because its hydrodynamic attributes (e.g. flow velocity, bottom shear stress) were spatially heterogeneous as a result of the geomorphic complexity of the floodplain (Ward et al. 1999; Amoros and Bornette 2002; Opperman et al. 2010). This result contrasts with the findings of other floodplain studies, most of which were conducted in large lowland river floodplains (Gomes et al. 2012; Bozelli et al. 2015; Larsen et al. 2019). Floods in lowland floodplains are typically characterized by slow-flowing, prolonged inundation periods that homogenize environmental conditions within the floodplain by connecting different water bodies with the main river and enabling mixing (Junk et al. 1989). This is in stark contrast with more dynamic sub-alpine rivers like the Maggia, where floods are short pulses with spatially heterogeneous effects (e.g. patterns of substrate scouring and deposition; Tockner et al. 2000). For instance, Malard et al. (2000) found that environmental heterogeneity increased during floods in the dynamic alpine floodplain of the Val Roseg, and Larsen et al. (2019) found no evidence of decreased environmental heterogeneity after floods in the Tagliamento River floodplain, a large Alpine river with a dynamic flow regime.

In contrast to the natural system, environmental heterogeneity increased over time in the experiment. This result made sense because the ponds were all excavated on the same day, and therefore local conditions (e.g. primary productivity) were rather similar early on in the experiment. Thus, the creation of the experimental ponds mimicked one of the seasonal inundation periods usually observed in large river floodplains. These inundation events homogenize floodplain habitats and communities, similar to what we observed in the experiment ponds (Tockner et al. 2000; Thomaz et al. 2007; Bozelli et al. 2015)). Gradually, differences in HC and gravel-bar specific hydrologies may have generated the divergence in environmental conditions, as reported elsewhere (e.g., Amoros & Bornette 2002). This contention is supported by the strong correlation between pond positioning within gravel bars and environmental conditions. It is also consistent with our general understanding of floodplain habitat dynamics, where environmental heterogeneity increases during times of hydrological isolation (Brunke et al. 2003; Thomaz et al. 2007; Starr et al. 2014).

### Ecological convergence results from different processes

A key finding in our study was that there was no divergence in community structure among habitats during succession. This contradicted H2 and was in stark contrast with the general expectation that habitat conditions and communities become more dissimilar among floodplain habitats during hydrological isolation (Thomaz et al. 2007; Starr et al. 2014; Bozelli et al. 2015; Larsen et al. 2019; Dong et al. 2020). In the natural system, communities and environmental conditions were more heterogeneous at early successional stages, but species-environment linkages were loose or absent, as indicated by the low environmental signal in the Mantel tests. Over time since the flood, environmental conditions became more similar, likely influenced by different degrees of HC, different water origins and the receding influence of the disturbance. Communities also slightly converged structurally as they reorganized along re-formed environmental gradients (as shown by the increasing environmental signal in the Mantel test). Together, these results suggest that the flood affected local communities in a stochastic manner and thus initially disrupted environment-biota linkages. Other studies have reported similar increased stochasticity in community structuring following floods, but with decreasing beta diversity resulting from the homogenizing effect of inundation periods (e.g. Thomaz et al. 2007; Bozelli et al. 2015). Here however, the flood temporarily increased stochasticity in communities but also increased beta diversity, likely because it acted more as a spatially heterogeneous disturbance event than as an event enabling strong connectivity (Ward et al. 1999; Tockner et al. 2000). The decrease in beta deviations may thus reflect an increasingly homogeneous selection effect driven by converging environmental conditions, rather than a shift towards more stochastically organized assemblages.

Contrary to the predictions of H2, beta diversity was initially high in the experiment, and the strong environmental effect in the Mantel tests at early dates shows that this resulted from early selection of species along a forming environmental gradient, rather than priority effects (Chase 2003; Chase and Myers 2011). Gradually, the role of dispersal gained importance and species-environment linkages weakened, despite increasing divergence in local environmental conditions. This result challenges the view that selection dominates in more mature communities (Jenkins 2006; Larsen and Ormerod 2014), rather it suggests that inherent time requirements associated with dispersal and reproduction caused a delayed occurrence of mass effects (e.g., for Chironomidae) that occulted other metacommunity processes (Mouquet and Loreau 2003; Van De Meutter et al. 2007; Tonkin et al. 2016). We argue that the increasing importance of the spatial signal is caused by mass effects rather than dispersal limitation because of the small spatial scale and the absence of dispersal barriers (Mouquet and Loreau 2003; Van De Meutter et al. 2007; Tonkin et al. 2016). It is important to consider that beta diversity would have initially increased between the construction of the ponds and the first sampling date, given that all communities had few individuals immediately following habitat creation. However, if the experiment had been run longer (i.e. past 45 days) the effects of mass effects may have dissipated as taxa, with lower dispersal potential eventually colonized the ponds, thus potentially enabling selection to dominate community assembly until the next reset by a natural disturbance (e.g. a flood). Similarly, if the experimental habitat creation had begun earlier in synchrony with the emergence of univoltine aquatic insects, selection may have dominated community assembly during the experiment.

In summary, the flood acted as a spatially heterogeneous disturbance and created heterogeneity in habitats and communities, and this initial stochasticity later receded as deterministic selection gained importance. In contrast, simultaneous pond construction meant that local conditions could only diverge over time in the experiment, but the small spatial scale facilitated mass effects caused a moderate convergence in community composition.

### Selection as the dominant metacommunity process, and varying importance of dispersal depending on context

The HMSC framework was used to examine the relative importance of three metacommunity processes (selection, dispersal and drift) averaged overtime. Considering that stochasticity is known to play a major role in community structuring in floodplains (Thorp et al. 2008; Devercelli et al. 2016), the total amount of explained variation (0.33 for the natural system and 0.39 for the experiment) remained within the range of other studies of community structuring in floodplain streams (Fernandes et al. 2014; Tonkin et al. 2016). The fact that the explanatory power of the models changed when removing or adding categories of predictors (environment, space and previous abundances), and that the SPE model had the highest explanatory power in both systems, suggested that each category had some influence on community composition and conveyed different information. Lastly, the comparison between the SE and SE_full_ models showed only minor differences in explanatory power and results, indicating that final interpretation on the importance of processes was little affected by the removal of the first sampling dates in the SPE model (Little and Altermatt 2018).

As predicted (H3), selection was the dominant process among the three processes examined and was stronger in the natural system than in the experiment. This is in line with the observation that selection tends to be the primary structuring process in floodplains (Urban 2004; Starr et al. 2014; Hill et al. 2017) and more generally in freshwaters (Heino et al. 2015). In the natural system, the negative associations between the upstream tip of gravel bars and the abundances of nine taxa suggest that high-HC habitats were favourable to the majority of observed taxa. It is quite likely that this strong effect results from unmeasured environmental variation (e.g. small variations in current velocity or deposited fine organic material) rather than simply the positioning of the habitat within the gravel bar. Despite the fact that most taxa favoured high HC habitats, Corixidae, Ceratopogoniidae and *Ephemerella* spp. appeared to favour low-DO environments. Finally, the fact that abundances of Baetidae, Corixidae and Dytiscidae were positively associated with the time since the flood suggested that these were likely late colonists in the pond system. In the experiment, chlorophyll-a and DO concentrations had the most influence on the community, thus also suggesting that environmental selection was largely constrained by the degrees of vertical HC, like in the natural system. Overall, we did not observe the expected clear patterns that sensitive rheophilic taxa, such as most EPT, would be more abundant in high-HC environments, whereas Coleoptera, Hemiptera and Diptera would prefer low-HC environments. Instead, the environmental effect on the community was largely shared among several variables, and this could be due to the moderate degree of collinearity observed between these variables.

The second metacommunity process, dispersal, had a negligible effect in the natural system but was more important in the experiment. Spatial structuring in metacommunities may result from dispersal limitations or mass effects, depending on the spatial context and the dispersal ability of taxa. And dispersal limitation is more prevalent at larger spatial scales and at lower degrees of ecological connectivity (Thompson and Townsend 2006b; Cañedo-Argüelles et al. 2015; Heino et al. 2015; Datry et al. 2016). Here, all taxa were capable of producing aerial adults capable of overland dispersal, and while there was some variation in their dispersal abilities, the spatial distances separating aquatic habitats were short compared with other studies where dispersal limitation was observed (Thompson and Townsend 2006b; De Bie et al. 2012; Gauthier et al. 2021). For instance, Tonkin et al., (2016) found no evidence of dispersal limitation in the structuring of a floodplain macroinvertebrate metacommunity (spatial scales of several hundred meters), and Gauthier et al. (2020) found that dispersal limitation was the primary structuring process at the scale of headwater stream networks (several kms). In addition, sampling was conducted in summer, a time of the year when all these taxa are likely to produce flying adults. We therefore argue that dispersal limitation was less likely to have caused the moderate spatial signal observed in our study.

Instead, considering the very small distances between experimental ponds, it is much more likely that mass effects occurred and caused this spatial structuring. Dispersal between experimental ponds may have also occurred via hyporheic flow paths, which might alternatively explain the mass effects we observed and also account for a portion of the selection effect attributed to HC—related environmental differences. It is possible that the more strongly connected ponds not only differed in terms of their environmental conditions, but also in terms of the colonization they received from the surrounding environment, via hyporheic flow paths (Wood et al. 2010; Vander Vorste et al. 2016).

The fact that previous abundances of the most abundant taxa explained around 25% of variation in community composition in both systems suggested that biotic interactions played an important role. While it is beyond the scope of this study to analyse each interaction in detail, the fact that abundances of three taxa in the natural system, and one taxon in the experiment, were positively affected by their previous abundance suggests that priority effects likely occurred (Little and Altermatt 2018). All putative interactions should be considered with caution because they could result from unmeasured environmental variation. But overall, taxa abundances seemed to be influenced by the previous abundances of several other taxa (Fig. S1.7 and S1.8), suggesting the occurrence of density-dependent selection and biotic interactions in general (Vellend 2010; Cadotte and Tucker 2017). The large influence of putative biotic interactions suggests that density-dependent selection may be more important to metacommunity structuring than is often assumed (García-Girón et al. 2019). For instance, Cadotte and Tucker (2017) and Cottenie and De Meester (2004) suggested that most selection in nature may result to a large degree from biotic interactions rather than pure environmental filtering.

## Conclusions

We showed that there were similarities between the two systems, which made their comparison meaningful. There were also key differences in spatial scales, HC and disturbance history that resulted in different dynamics of habitats and communities. We showed that the flood acted as a spatially heterogeneous disturbance that increased beta diversity, and that spatial scale and flood heterogeneity determine the relative importance among structuring processes during ensuing ecological convergence (Urban 2004; Vanschoenwinkel et al. 2010; Vanschoenwinkel et al. 2013; Leibold and Chase 2018). Our study helps to explain the drivers of community assembly in riverine floodplain environments, which is essential for managing trade-offs between biodiversity and societal demands for freshwater resources in the face of climate change.

This study also highlighted the need for future research. First, our experimental and natural systems differed in various structural aspects (e.g. spatial scale, disturbance, age of communities); therefore, future work should focus on isolating the effects of each of these factors on metacommunity structuring. Second, we showed that biotic interactions may indeed have a prominent role in structuring communities in floodplains (Urban 2004; Paillex et al. 2013). Considering the central role of biotic interactions in selection (Cottenie and De Meester 2004; Cadotte and Tucker 2017), identifying their determinants and consequences may greatly improve our ability to explain and predict metacommunity structuring (García-Girón et al. 2019). Finally, expanding on Tockner, Malard and Ward's work (2000), we argue that the response of environmental heterogeneity and biodiversity to floods may differ between large river floodplains and more dynamic floodplains. Therefore, characterizing the factors that drive these differences would be very beneficial to our understanding of floodplains in general and their management.

## Supplementary Information

Below is the link to the electronic supplementary material.

Supplementary file 1 (DOCX 1191 KB)

## Data Availability

The data that support the findings of this study are available from the corresponding author upon reasonable request.
