# Supplementary material for: Convergence in floodplain pond communities indicates different pathways to community assembly
Source: Aquat Sci. 2023 Mar 31;85(2):59. doi: 10.1007/s00027-023-00957-9 (PMC10066089; doi:10.1007/s00027-023-00957-9)
Supplement: Supplementary file 1 — (DOCX 1191 KB) [file 27_2023_957_MOESM1_ESM.docx]

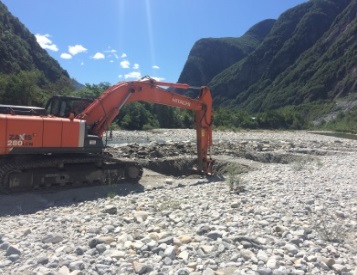


Figure S1.1 Creation of experimental ponds in the Maggia river floodplain

Figure S1.2 Biplot showing the contribution of environmental variables to the first two PCA axes for the experiment. The measured variables were water temperature (°C), pH, POM (g/m^2^), silicate concentration (mg/l), nitrate concentration (mg/l), dissolved oxygen (mg/l), chlorophyll-a (µg /L), conductivity (µS/cm), and pond volume (m^3^).

Figure S1.3 Biplot showing the contribution of environmental variables to the first two PCA axes for the experiment. The measured variables were water temperature (°C), dissolved oxygen (mg/l), pH, POM (g/m^2^), silicate concentration (mg/l), nitrate concentration (mg/l), chlorophyll-a (µg /L), conductivity (µS/cm), and pond volume (m^3^), substrate diversity and D50 (cm).

Figure S1.4. GAMM model showing the relationship between PCA1 in the experiment, the distance between the experimental ponds and the upstream tip of the gravel bars, and the Y coordinate representing the longitudinal position of each habitat along the floodplain.

Figure S1.5. GAMM model showing the relationship between PCA1 in the experiment and the days elapsed since the construction of the ponds.

Figure S1.6. GAMM model showing the relationship between PCA1 in the natural system, the distance between the aquatic habitats and the upstream tip of the gravel bars, and the Y coordinate representing the longitudinal position of each habitat along the floodplain.

Figure S1.7. Results of the HMSC model showing the proportion of variation explained by the previous abundances of the most abundant taxa, for the natural system.

Figure S1.8. Results of the HMSC model showing the proportion of variation explained by the previous abundances of the most abundant taxa, for the experiment.

Table S1.1 Contribution of environmental variables to the first two axes of the PCA.

| **System** | **Variable** | **PC1** | **PC2** |
| --- | --- | --- | --- |
| **Experiment** | Proportion explained (%) | 34.0 | 23.2 |
|  | Conductivity (µS/cm) | 1.472 | -0.461 |
|  | pH | 0.986 | 0.9255 |
|  | Nitrate (mg/L) | -0.075 | -0.627 |
|  | Silicate (mg/L) | 1.131 | 1.059 |
|  | DO (mg/L) | -1.216 | 0.895 |
|  | Temperature (°C) | -0.466 | -1.456 |
|  | Chlorophyll a (µg /L) | 1.441 | -0.191 |
|  | Volume (m^3^) | 0.968 | -0.822 |
| **Natural** | Proportion explained (%) | 35.8 | 21.7 |
|  | DO (mg/L) | 1.410 | -0.189 |
|  | Temperature (°C) | -0.769 | 0.418 |
|  | Conductivity (µS/cm) | -1.086 | 0.976 |
|  | pH | 0.144 | -0.274 |
|  | Nitrate (mg/L) | 1.394 | -0.304 |
|  | Silicate (mg/L) | 0.277 | -1.298 |
|  | Substrate diversity | 1.305 | 0.652 |
|  | Substrate D50 (cm) | 0.827 | 1.003 |
|  | CPOM (g/m^2^) | -0.651 | -1.054 |
|  | AFDM (g/m^2^) | -0.763 | -0.138 |

Table S1.2 Relationship between the first two PCA axes with spatial and temporal variables.

| **System** | **Response** | **Predictors** | **edf** | **t (F) - value** | **p value** | **R^2^ _adj_** |
| --- | --- | --- | --- | --- | --- | --- |
| **Experiment** | PC1 | Y | 1.93 | 48.3 | <0.001 | 0.86 |
|  |  | Distance upstream | 1.7 | 1.93 | <0.001 |  |
|  |  | Date | 2.66 | 111.8 | <0.001 |  |
|  | PC2 | Y | 1.95 | 12.4 | <0.001 | 0.77 |
|  |  | Distance upstream | 1.67 | 23.13 | <0.001 |  |
|  |  | Date | 2.5 | 44 | <0.001 |  |
| **Survey** | PC1 | Y | 2.89 | 6.04 | 0.001 | 0.79 |
|  |  | Date | 2.05 | 0.84 | 0.34 |  |
|  |  | Distance upstream | 2.9 | 65.57 | <0.001 |  |
|  | PC2 | Y | 2.6 | 33.27 | <0.001 | 0.63 |
|  |  | Date | 1 | 5.24 | 0.02 |  |
|  |  | Distance upstream | 1 | 3.68 | 0.06 |  |

Table S1.3. Average values for environmental variables

| **Variable** | **System** | **Mean value** |
| --- | --- | --- |
| DO (mg/L) | Experiment | 8.48 |
|  | Natural | 7.74 |
| Temperature (°C) | Experiment | 19.09 |
|  | Natural | 17.27 |
| Conductivity (µS/cm) | Experiment | 79.25 |
|  | Natural | 68.55 |
| pH | Experiment | 7.10 |
|  | Natural | 6.90 |
| Nitrate (mg/L) | Experiment | 0.48 |
|  | Natural | 0.50 |
| Silicate (mg/L) | Experiment | 7.90 |
|  | Natural | 10.16 |
| Chlorophyll a (µg /L) | Experiment | 103.98 |
|  | Natural | 0.00 |
| CPOM (g/m2) | Natural | 1.88 |
| Substrate D50 (cm) | Natural | 6.34 |
| Substrate diversity | Natural | 1.27 |
| Volume (m3) | Experiment | 606.87 |

Table S1.4. Results for the comparison of different HMSC models. The SPE model includes spatial, environmental and biotic (abundances of the most abundant early colonists) explanatory variables, SE includes the spatial and environmental variables, SP includes the spatial and biotic variables and S only the spatial variables. The SE_full_ is the SE model run on all four dates to verify that removing the first dates in the other analyses did not drastically change the environmental and spatial effects on populations.

| **HMSC Model** | **System** | **Mean R^2^** |
| --- | --- | --- |
| SPE | Experiment | 39.25 |
|  | Natural | 33.43 |
| SE | Experiment | 32.80 |
|  | Natural | 24.40 |
| SP | Experiment | 28.02 |
|  | Natural | 16.73 |
| S | Experiment | 3.50 |
|  | Natural | 21.16 |
| SE_full_ | Experiment | 30.50 |
|  | Natural | 0.23 |

| Explanatory variable | Taxon | Mean posterior estimate |
| --- | --- | --- |
| Dist. Upstream | Ceratopogoniidae | -0.004 |
| Dist. Upstream | Corixidae | -0.003 |
| Dist. Upstream | Elmidae | -0.003 |
| Dist. Upstream | *Ephemerella* spp. | -0.004 |
| Dist. Upstream | *Habropelptoides* spp. | -0.003 |
| Dist. Upstream | Heptageniidae | -0.003 |
| Dist. Upstream | *Leuctra* spp. | -0.002 |
| Dist. Upstream | polycentropodidae | -0.003 |
| Dist. Upstream | Tipulidae | -0.002 |
| DO | Ceratopogoniidae | -0.378 |
| DO | Corixidae | -0.260 |
| DO | *Ephemerella* spp. | -0.175 |
| DO | Hydroptilidae | 0.213 |
| Chlorophyll a. | Corixidae | -0.007 |
| Temperature | Corixidae | 0.116 |
| Dissolved silicate | Corixidae | 0.149 |
| Dissolved silicate | Dytiscidae | 0.246 |
| Dissolved silicate | Hydroptilidae | -0.166 |
| Time | Baetidae | 0.014 |
| Time | Corixidae | 0.022 |
| Time | Dytiscidae | 0.008 |
| Prev. Abund. Tipulidae | Tipulidae | 0.172 |

Table S1.5. Results of the HMSC model for the natural system, showing the mean of the posterior estimate for the association between the explanatory variables and the response taxa. Only strong effects are reported here, corresponding to associations where the 95% confidence interval for this particular association is non-overlapping with 0.

Table S1.6. Results of the HMSC model for the experiment, showing the mean of the posterior estimate for the association between the explanatory variables and the response taxa. Only strong effects are reported here, corresponding to associations where the 95% confidence interval for this particular association is non-overlapping with 0.

| Explanatory variable | Taxon | Mean posterior estimate |
| --- | --- | --- |
| Chlorophyll a. | Culicinae | 0.002 |
| Chlorophyll a. | *Ephemerella* spp. | -0.009 |
| Chlorophyll a. | *Laccobius* spp. | 0.007 |
| Chlorophyll a. | Nemouridae | -0.006 |
| Dist. Upstream | Culicinae | 0.006 |
| Dist. Upstream | *Leuctra* spp. | 0.010 |
| DO | Baetidae | 0.547 |
| DO | Ceratopogoniidae | 0.456 |
| DO | Chironomidae | -0.414 |
| DO | Hydroptilidae | -0.397 |
| Nitrate concentration | Hydroptilidae | 7.797 |
| Nitrate concentration | *Laccobius* spp. | -11.098 |
| Prev. Abund. Baetidae | Baetidae | 0.269 |
| Prev. Abund. Baetidae | *Leuctra* spp. | -0.393 |
| Prev. Abund. Culicinae | Culicinae | 0.262 |
| Prev. Abund. Culicinae | Hydroptilidae | -0.217 |
| Prev. Abund. Culicinae | *Laccobius* spp. | 0.365 |
| Prev. Abund. Tanypodinae | Culicinae | -0.209 |
| Prev. Abund. Tanypodinae | Hydroptilidae | 0.271 |
| Prev. Abund. Tipulidae | Baetidae | 0.274 |
| Prev. Abund. Tipulidae | Tipulidae | 0.141 |
| Silicate concentration | Nemouridae | 1.138 |
| Temperature | Culicinae | -0.205 |
| Temperature | Hydroptilidae | -0.292 |
| Time | Hydroptilidae | -0.050 |
